# Genetic Deletion of Akt3 Induces an Endophenotype Reminiscent of Psychiatric Manifestations in Mice

**DOI:** 10.3389/fnmol.2017.00102

**Published:** 2017-04-10

**Authors:** Yan Bergeron, Geneviève Bureau, Marie-Élaine Laurier-Laurin, Eric Asselin, Guy Massicotte, Michel Cyr

**Affiliations:** Department of Medical Biology, Université du Québec à Trois-RivièresTrois-Rivières, QC, Canada

**Keywords:** Akt3, GSK3α/β, lithium, behavior, anxiety, depression

## Abstract

The protein kinase B (PKB/Akt), found in three distinctive isoforms (PKBα/Akt1, PKBβ/Akt2, PKBγ/Akt3), is implicated in a variety of cellular processes such as cell development, growth and survival. Although Akt3 is the most expressed isoform in the brain, its role in cerebral functions is still unclear. In the present study, we investigated the behavioral, electrophysiological and biochemical consequences of Akt3 deletion in mice. Motor abilities, spatial navigation, recognition memory and LTP are intact in the Akt3 knockout (KO) mice. However, the prepulse inhibition, three-chamber social, forced swim, tail suspension, open field, elevated plus maze and light-dark transition tests revealed an endophenotype reminiscent of psychiatric manifestations such as schizophrenia, anxiety and depression. Biochemical investigations revealed that Akt3 deletion was associated with reduced levels of phosphorylated GSK3α/β at serine 21/9 in several brain regions, although Akt1 and Akt2 levels were unaffected. Notably, chronic administration of lithium, a mood stabilizer, restored the decreased phosphorylated GSK3α/β levels and rescued the depressive and anxiety-like behaviors in the Akt3 KO mice. Collectively, our data suggest that Akt3 might be a critical molecule underlying psychiatric-related behaviors in mice.

## Introduction

Psychiatric illnesses such as depression, bipolar disorders, anxiety and schizophrenia represent an important public health problem causing not only personal suffering but also a tremendous economic burden (Wittchen et al., 2011; Vigo et al., 2016). Over the recent years, many studies have highlighted the role of genetic variations, abnormal neural development and dysregulation of neurotransmitters to explain the development of these disorders (Renoir, 2014). Even if these discoveries have led to a plethora of pharmacological treatments, their efficacy is still limited (Gould and Einat, 2007; Duman and Voleti, 2012). Therefore, identification of key cell signaling molecules and pathways are critical to uncover novel therapeutic targets and successful clinical interventions.

A family of serine/threonine kinases, the protein kinase B (PKB/Akt), has received extensive consideration in recent years for its possible involvement in psychiatric conditions. For instance, Akt activity is known to be modified by antidepressants, antipsychotics and mood stabilizers (Beaulieu et al., 2009). The Akt protein is constituted by three isoforms termed Akt1/PKBα, Akt2/PKBβ and Akt3/PKBγ. Despite the fact that they are showing strong homologies, these isoforms are encoded by distinct genes (Diez et al., 2012). The most abundant isoforms is Akt1. Akt1 is ubiquitously expressed whereas Akt2 is primarily expressed in insulin-responsive tissues and Akt3 is very much expressed in brain and testes (Hers et al., 2011). Understanding brain Akt functions has been gained by the generation of transgenic and knockout (KO) mouse models (Yang et al., 2004; Dummler et al., 2006). For instance, a partial lethality is observed in mice lacking Akt1 and the surviving mice are reduced in size at birth and remain small throughout life (Cho et al., 2001; Yang et al., 2003). Akt2 is particularly important for glucose metabolism (Cho et al., 2001; Garofalo et al., 2003) and a role in photoreceptor survival is observed (Li et al., 2007). Recently, signs of anxiety and depressive-like behaviors have been reported in mice lacking Akt2 (Leibrock et al., 2013). The role of Akt3 is less clear even though it appears to be the most highly expressed isoform in the brain. Mice lacking Akt3 exhibit small brains (Easton et al., 2005; Poduri et al., 2012). It is noteworthy that a multi-stage schizophrenia genome-wide association study has identified Akt3 as a potential contributor to schizophrenia, which underscore the importance of investigating the role of Akt3 in the brain (Schizophrenia Working Group of the Psychiatric Genomics Consortium, 2014).

The goal of the present study is to define the behavioral, electrophysiological and biochemical consequences of Akt3 deletion in mice. Our findings reveal that Akt3 deletion in mice does not affect motor, cognitive and synaptic functions, but is associated with behavioral abnormalities reminiscent of psychiatric symptoms. Notably, the depressive and anxiety-like behaviors are reversed by a chronic lithium treatment. Our data provide evidence that Akt3 signaling may play a role in the pathogenesis of psychiatric conditions.

## Materials and Methods

### Animals

This study was carried out in accordance with the recommendations of the UQTR Institutional Animal Care and Use Committee and performed in accordance with the Canadian Council on Animal Care. The generation of Akt3 KO mice has been described previously (Easton et al., 2005). In all experiments, 12–18 weeks-old Akt3 KO male mice (C57bl/6j background) and their respective wild type (WT) littermates were used. They were housed in groups of 3–4 per cage (12 h light-dark cycles) with *ad libitum* food and water. Akt3 KO and WT mice were genotyped by PCR analysis of DNA samples obtained from ear punches. Five sets of experiments were conducted on five independent cohorts of WT and Akt3 KO mice. (1) One set of experiments involved behavioral testing run from least to most invasive, to decrease the chance that behavioral responses are altered by prior test. Morris water maze, novel object recognition, elevated plus maze, light-dark transition, three-chamber social test, forced swim test (FST) and tail suspension test (TST) were performed in this sequence in drug naïve WT and Akt3 KO mice. (2) Another set investigates, in order, the rotarod, wire suspension, pole and stepping tests. (3) The open field and prepulse inhibition tests were performed at the Neurophenotyping Centre (Douglas Mental Health Institute, Montreal, QC, Canada). (4) One set included drug naïve WT and Akt KO mice exclusively for electrophysiology experiments. Finally, (5) one set investigated the elevated plus maze and FSTs in four groups of mice: WT-Standard chow, WT-Lithium, Akt3 KO-Standard chow and Akt3 KO-Lithium.

### Behavioral Tests

To perform comprehensive behavioral analyses of WT and Akt3 KO mice, we used previously well-characterized tests (Crawley, 2008). A period of 30 min of habituation in the experimental room was allowed prior the test. All tests were performed between 8 a.m to 4 p.m and were separated from each other by 2 days at least.

#### Motor Ability Tests

General motor behavior of mice was evaluated using the rotarod, the wire suspension, pole and stepping tests as we described previously (Chagniel et al., 2014). First, mice were pre-trained on the rotarod (AccuScan Instruments, Columbus, OH, USA) five times at a constant rate of 10 rpm. A resting time of 180 s was allowed between each trial. The end of a trial was considered when mice were falling off the rod or when they reached 150 s. The next day following the pre-training, mice performed the rotarod three times at a constant rate of 10 rpm and latency to fall was recorded for each trial. Briefly, the wire suspension test consisted to hang the animal with its paws on the middle of a wire fixed horizontally between two platforms (length: 80 cm, height: 25 cm). The time needed to reach one platform was recorded. For the pole test, mice were placed at the upper end of a pole (length: 50 cm, diameter: 1.5 cm). The time taken to turn down and reach the base of the pole was recorded. A maximum time of 120 s was allowed to execute the wire suspension and pole test. The stepping test consists to lift up the mouse’s hind legs by pulling up the tail, leaving only the forepaws on the table. Then, the experimenter pulled the animal backward by the tail (1 min in 5–6 s), until the other edge of the table was reached. Each trial was recorded using a video camera and the number of adjusting steps for both forepaws was calculated.

#### Morris Water Maze

In a circular pool (122 cm of diameter) filled with opaque water (23°C), mice learning and memory was evaluated. For spatial acquisition, mice were trained to reach the submerged platform (12 × 10 cm) four times per day for five consecutive days. Latency to find the platform was measured. On the 6th day, a probe trial was given. The platform was withdrawn from the pool and the time of exploration in each quadrant was measured.

#### Novel Object Recognition

Mice were habituated 10 min in the test box (40 × 40 cm) for three consecutive days. On day 4, two identical objects (dice) were placed in the opposite corners of the box and mice were allowed to explore for 10 min. Twenty-four hours later, one of the familiar objects was replaced by a novel object (red ball) and mice were left in the box exploring for 10 min. The session was recorded using a video camera and exploration of the familiar or the novel object was evaluated. Exploration of the object was considered when a mouse displayed any investigative behaviors (head toward object, sniffing closely or entering an area within 1 cm around the object).

#### Prepulse Inhibition

Prepulse inhibition (PPI) was assessed in a sound-attenuated chamber (8 × 16 cm). The session began with a 5 min acclimation to a background noise of 70 dB. This is followed by a 10 trials block of a 120 dB startle stimulus (without prepulse). Then, to assess PPI, the startle stimulus was preceded by a 30 ms prepulse stimulus. The intensity of the prepulse varied between 73 and 85 dB, in increment of 3 dB. Each intensity intervals were presented five times and were randomly generated. The inter-trial intervals were variable from 5 s to 30 s with an average of 15 s. Startle responses were collected every 1 ms for 100 ms after startle stimulus and were averaged for each prepulse intensity. Percentage of prepulse inhibition was calculated as: [(startle + prepulse)/startle) × 100].

#### Three-Chamber Social Test

Mice were tested for social interactions using a three-chamber box (20 × 40 cm, each chamber). First, the mouse was placed in the central chamber and was allowed to explore the entire box for a 5 min habituation session. In the session I, which evaluate sociability, the mouse was given the choice to interact with either an empty wired cage (9 cm of diameter, 11 cm height, placed in one side chamber) or an unfamiliar mouse (stranger 1) placed in a similar cage on the other side chamber. For 10 min, the tested mouse was allowed to explore and the time spent exploring the empty cage and the stranger I was recorded. In the session II, which test the preference for social novelty, a second unfamiliar mouse (stranger II) was placed in the empty chamber. The time spent exploring the stranger I or the stranger II was recorded for 10 min.

#### Forced Swim Test

Mice were deposed individually into a transparent plastic cylinder (20 cm height, 15 cm diameter), filled with water (11 cm deep, kept at 25°C). Immobility time (no limb movement) was measured in the last 4 min of the 6 min recorded session.

#### Tail Suspension Test

Mice were suspended by the tip of the tail 35 cm to the ground for a period of 6 min. Immobility time was measured on the last 4 min of the test. Mice were considered immobile when they ceased struggling and remained motionless.

#### Open Field

Mice were placed 60 min in a box (1 × 1 m) for habituation. After habituation, the time spent in the central zone were recorded and measured for 90 min with Versamax software (Accuscan Instruments, Columbus, OH, USA).

#### Elevated Plus Maze

The elevated plus maze consisted in a cross-shaped platform with two open and two closed arms, elevated 50 cm above the floor. Mice were placed in the center of the maze facing one of the closed arms. Mice were allowed to explore the cross-shaped platform for 10 min. The number of entries as well as the time spent in the open and closed arms were measured.

#### Light-Dark Transition

The apparatus consisted in a box (40 × 40 cm) divided into two equal sections. One chamber was brightly illuminated, whereas the other chamber was dark. Mice were placed into the dark area and allowed to move freely between the two chambers through an open door for 10 min. The total number of transitions from the dark to the light chamber and the time spent in the light chamber were recorded.

### Chronic Lithium Treatment

For chronic lithium treatment (LiCl), mice were placed on a 0.2% LiCl diet (Harlan Teklab, Madison, WI, USA) for 5 days followed by a 0.4% LiCl diet for an additional 20 days (O’Brien et al., 2004). All mice received drinking water supplemented with 0.9% saline to reduce ion imbalance caused by polyurea of lithium treatment. Control group mice were fed with standard chow. Behavioral experiments began on day 10. At day 26, mice were anesthetized under isoflurane, decapitated and brains were immediately removed. At the same time, blood was collected through a cardiac puncture for lithium serum dosage. Lithium levels in the serum samples were measured with a flame photometer (Perkin-Elmer, model Analyst 100). The hippocampus, striatum, anterior cortex and cerebellum were dissected rapidly and frozen on dry ice until processed for western blot analysis.

### Biochemical Analysis

WT and Akt3 KO mice were anesthetized under isoflurane, decapitated and brains were immediately removed. The striatum, hippocampus, cerebellum and anterior cortex were dissected, frozen on dry ice and preserved at −80°C. The western blots were performed as previously described (Bergeron et al., 2014). Antibodies used were raised against Akt1, Akt2, Akt3, phosphorylated GSK3α/β at serine 21/9 (Cell signaling, 1:1000), total GSK3α/β (Millipore, 1:2000) and GAPDH (Abcam, MA, USA 1:20,000). Membranes were washed and incubated with appropriate HRP-conjugated secondary antibody (Cell signaling, 1:5000). To visualize protein bands, chemiluminescence reactions were used (SuperSignal West Femto chemiluminescence kit, Pierce Chemical Co, IL, USA). Densitometry analysis was performed using the VisionWorks LS software (UVP Bioimaging Upland, Upland, CA, USA) and expressed as relative optical density.

### Electrophysiology

Hippocampal slice preparation was performed as described before (Laurier-Laurin et al., 2014). Briefly, WT and Akt3 KO mice were anesthetized under isoflurane, decapitated and hippocampi were dissected in an ice-cold cutting buffer. Transverse slices (350 μm) were prepared with a McIlwain tissue chopper and transferred in artificial cerebrospinal fluid. The slices were allowed to recover 1 h before recordings. A glass recording electrode (1–5 MΩ filled with 2 M NaCl) was positioned in the stratum radiatum of area CA_1_ of the hippocampus to record field excitatory postsynaptic potentials (fEPSPs) evoked by a bipolar electrode (twisted 60 μm nichrome), activating fibers of the Schaffer collateral system. Stimulation consisted of a 0.1 ms pulse delivered every 30 s (0.033 Hz) with current intensity adjusted to obtain 40%–50% of maximal fEPSPs. After a 10 min baseline period, theta-burst stimulations (10 bursts of four stimulation pulses at 100 Hz) were given at 200 ms (5 Hz) intervals. After this block of tetanic stimulation, fEPSPs were again collected at the baseline presentation rate (0.033 Hz). In all cases, fEPSPs were recorded with NAC 2.0 software (Theta Burst Corp., Irvine, CA, USA) and the response to stimulation was quantified by calculating the initial amplitude of resulting fEPSPs.

### Statistical Analysis

Data were analyzed with GraphPad Prism software (version 5.0, Graph Pad Software, San Diego, CA, USA). Statistical analysis were determined by unpaired Student’s *t*-test, two-way repeated-measures analysis of variance (ANOVA) followed by the Bonferroni *post hoc* test or by paired Student’s Wilcoxon *t*-test. Data are shown as the mean ± SEM and statistical significance was set at *P* < 0.05.

## Results

### Levels of Akt Isoforms in the Akt3 KO Mouse Brain

**Figure 1:**
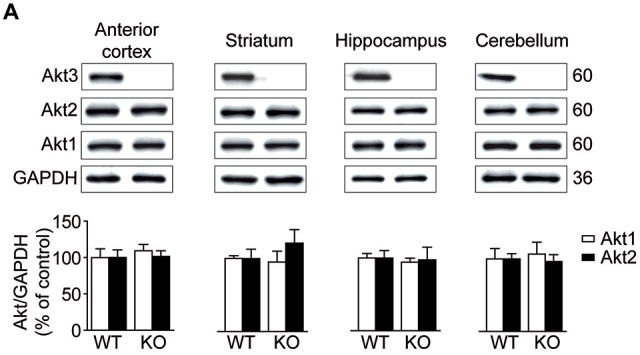
**Levels of Akt isoforms in the Akt3 knockout (KO) mouse brain. (A)** Levels of Akt1, Akt2 and Akt3 were assessed by western blot in the anterior cortex, striatum, hippocampus and cerebellum. The data, expressed relative to GAPDH, represent the mean of relative optical density of Akt1 and Akt2 (expressed as a percentage of control values) ± SEM, *n* = 3–4; triplicate experiments for each mouse/group. Since Akt3 protein was undetectable in Akt3 KO mice, we did not quantify the optical density.

Akt protein levels were assessed by Western blot in the anterior cortex, striatum, hippocampus and cerebellum. As expected, Akt3 protein was undetectable in brain regions examined in Akt3 KO mice (Figure 1A). To evaluate whether Akt1 or Akt2 expression was affected by the Akt3 deletion, levels of Akt1 and Akt2 were also measured. Compared to WT mice, levels of Akt1 and Akt2 remained unchanged in Akt3 KO brain structures considered (Figure 1A).

### Normal Motor Abilities and Cognitive Functions in Akt3 KO Mice

**Figure 2:**
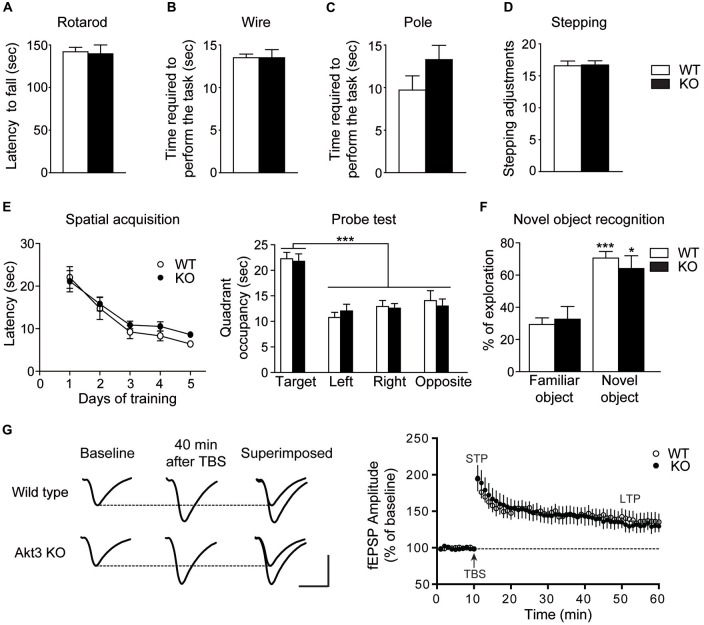
**Normal motor abilities and cognitive functions in Akt3 KO mice.** Motor abilities on the **(A)** Rotarod, **(B)** Pole, **(C)** Wire and **(D)** stepping tests of Akt3 KO and wild type (WT) mice were evaluated. Data represent the average mean latency to fall expressed in seconds from three assays (rotarod) ± SEM, *n* = 7 mice/group. Data represent the mean time require to perform the test (pole and wire) and the mean numbers of adjusting steps (stepping) ± SEM, *n* = 6–7 mice/group. **(E)** Mice were trained on the Morris water maze. Spatial acquisition is evaluated by the latency to reach the hidden platform at each day of training expressed in seconds ± SEM, *n* = 12–17 mice/group. Memory is evaluated during the probe trial by the time spent in each quadrant expressed in seconds ± SEM, *n* = 12–17 mice/group. ***p < 0.001 vs. target quadrant. **(F)** The novel object recognition test was performed to evaluate memory. Time spent exploring the familiar or the novel object is shown as a ratio of the total time spent exploring both objects ± SEM, *n* = 10–11 mouse/group. *p < 0.05, ***p < 0.001 vs. familiar object. **(G)** Examples of average fEPSPs elicited in the stratum radiatum before and after theta-burst stimulation in WT and Akt3 KO slices. Each sample trace is an average of five consecutive responses. Calibration bar: 5 ms; 1 mV. Amplitude of fEPSPs was measured before and after theta-burst stimulation and fEPSPs were expressed as the percentage of the average response before theta-burst stimulation (*arrow*). The values obtained during the period preceding theta-burst stimulation were averaged to derive baseline value. The data are expressed as percentages of baseline values and each point represents the mean ± SEM, *n* = 7.

To verify the effect of Akt3 deletion on general motor abilities, we performed well recognized motor tests: the rotarod, the pole, wire suspension and stepping tests. No difference was observed between Akt3 KO and WT performances in all tests, revealing that Akt3 KO mice do not suffer from a generalized deficit in locomotion, motor abilities and activity levels (Figures 2A–D).

To study the impact of Akt3 deletion on cognitive functions, we performed the Morris water maze and the novel object recognition tests. After 5 days of training in the Morris water maze, two-way repeated measures ANOVA followed by the Bonferroni *post hoc* test revealed that Akt3 KO and WT mice required similar time to find the hidden platform (genotype, *F*_(1,135)_ = 1.388; *p* > 0.05; days, *F*_(4,135)_ = 23.36; *p* < 0.001; genotype × days, *F*_(4,135)_ = 0.2930; *p* > 0.05; Figure 2E). During the probe test, Akt3 KO and WT mice spent more time in the target quadrant compared to other quadrants (genotype, *F*_(1,108)_ = 0.02350; *p* > 0.05; quadrant, *F*_(3,108)_ = 25.06; *p* < 0.001; genotype × quadrant, *F*_(3,108)_ = 0.2847; *p* > 0.05; Figure 2E). In the novel object recognition test, two-way ANOVA followed by the Bonferroni *post hoc* test revealed that both mice groups explored preferentially the novel object rather than the familiar object (genotype, *F*_(1,38)_ = 0.4278; *p* > 0.05; objects, *F*_(1,38)_ = 23.12; *p* < 0.001; genotype × objects, *F*_(1,38)_ = 1.254; *p* > 0.05; Figure 2F).

Adjacent coronal brain slices containing the hippocampus from Akt3 KO and WT mice were prepared for electrophysiological experiments. Theta-burst stimulation of the Schaffer commissural pathway in area CA1 of the hippocampus was then used to elicit synaptic potentiation. In the present investigation, evoked fEPSPs were statistically compared immediately and 40 min after theta-burst stimulation to determine the possible impact of Akt3 deletion on short-term and long-term potentiation, respectively. We found that neither short nor long-term potentiation was modified in Akt3 KO mice compared to WT mice (paired Student’s Wilcoxon *t*-test, *p* > 0.05; Figure 2G).

### Akt3 KO Mice Exhibit an Endophenotype Reminiscent of Psychiatric Conditions

**Figure 3:**
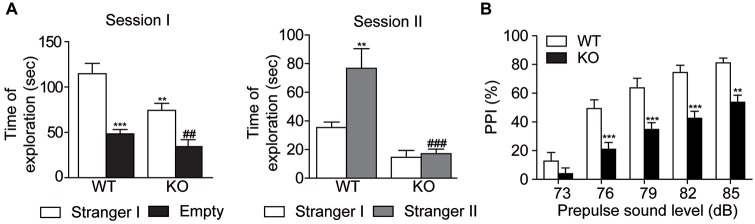
**Decreased social behaviors and impaired sensorimotor gating in Akt3 KO mice.** Social behavior and social novelty were examined in the three-chamber sociability test. **(A)** On session I, sociability was evaluated by the time Akt3 KO and WT mice were exploring the stranger mouse (stranger I) and the empty cage. Data represent the mean time of exploration expressed in seconds ± SEM, *n* = 8–11 mice/group. **p < 0.01, ***p < 0.001 vs. exploration of stranger mouse I by WT mice; ^##^*P* < 0.01 vs. exploration of stranger mouse I by Akt3 KO. On session II, preference for social novelty was evaluated by comparing the time mice explored the familiar stranger mouse I and the novel stranger mouse II. Data represent the mean exploration time expressed in seconds ± SEM, *n* = 8–11 mice/group. **p < 0.01 vs. exploration of stranger mouse I by WT mice, ^###^*p* < 0.001 vs. exploration of stranger mouse II by WT mice. **(B)** Sensorimotor gating was evaluated by measuring the level of prepulse inhibition for different prepulse intensities. Data represent the mean percentage ± SEM, *n* = 11–12 mice/group. **P < 0.01, ***P < 0.001 vs. WT mice.

Social behavior was evaluated by the three-chamber social test. In the Session I, two-way ANOVA followed by the Bonferroni *post hoc* test showed that Akt3 KO and WT mice explored preferentially the cage with a stranger mouse than an empty cage. Interestingly, Akt3 KO mice showed significantly lower investigation time of stranger mouse than WT mice, reflecting impaired social behavior (cage type, *F*_(1,34)_ = 38.18; *p* < 0.001; genotype, *F*_(1,34)_ = 10.09; *p* < 0.01; cage type × genotype, *F*_(1,34)_ = 2.361; *p* > 0.05; Figure 3A). In the Session II, the preference for social novelty was measured. WT mice spent more time exploring the novel stranger mouse II than the familiar stranger mouse I, while Akt3 KO mice did not show preference between stranger mouse I and II (cage type, *F*_(1,32)_ = 23.08; *p* < 0.05; genotype, *F*_(1,32)_ = 6.829; *p* < 0.001; cage type × genotype, *F*_(1,32)_ = 5.362; *p* < 0.05; Figure 3A). The PPI test was performed to evaluate sensorimotor gating. Two-way ANOVA followed by the Bonferroni *post hoc* test revealed an overall decrease in PPI responses in Akt3 KO mice compared to WT mice (intensity, *F*_(4,105)_ = 39.85; *p* < 0.001; genotype, *F*_(1,105)_ = 58.46; *p* < 0.001; intensity × genotype, *F*_(4,105)_ = 1.609; *p* > 0.05; Figure 3B).

**Figure 4:**
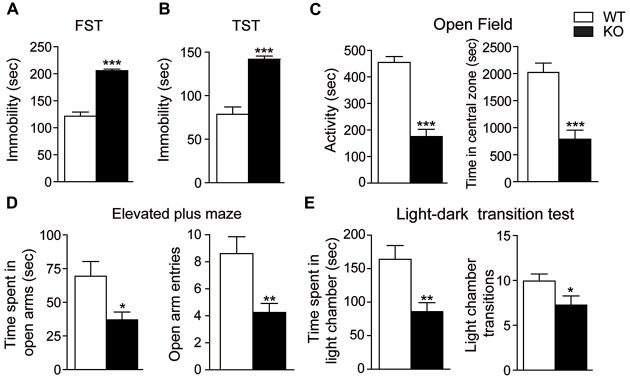
**Depressive and anxiety-like behaviors in Akt3 KO mice.** Depressive-like behaviors were evaluated by measuring the immobile time of Akt3 KO mice and WT mice in **(A)** the forced swim test (FST) and **(B)** the tail suspension test (TST). **(C)** In the open field, anxiety-like behavior was based on mice activity and distance traveled in the central zone. **(D)** In the elevated plus maze, time in open arms and number of entries in open arms were measured. **(E)** In the light-dark test, time spent in the light chamber and number of entries in the light chamber was measured. All data are expressed as the mean ± SEM, *n* = 11–17 mice/group. *p < 0.05, **p < 0.01, ***p < 0.001 vs. WT mice.

We next examined depressive-like behaviors in mice. In the forced swim and the TSTs, Akt3 KO mice spent more time immobilized compared to WT mice (unpaired *t*-test, *p* < 0.001; Figures 4A,B). Anxiety-related behaviors were also evaluated. The open field test revealed that Akt3 KO mice were less active (unpaired *t*-test, *p* < 0.001) and covered a shorter distance in the central zone compared to WT mice (unpaired *t*-test, *p* < 0.001; Figure 4C). The elevated plus maze showed that Akt3 KO mice spent a lower proportion of time in the open arms (unpaired *t*-test, *p* < 0.05) and entered less frequently in the open arms compared to WT mice (unpaired *t*-test, *p* < 0.01; Figure 4D). Finally, we observed in the light-dark transition test that Akt3 KO mice spent a lower proportion of time (unpaired *t*-test, *p* < 0.01) and exhibited a lower number of transitions in the lighted chamber compared to WT mice (unpaired *t*-test, *p* < 0.05; Figure 4E).

### Chronic Lithium Treatment Rescued Depressive and Anxiety-Like Behaviors Observed in Akt3 KO Mice

**Figure 5:**
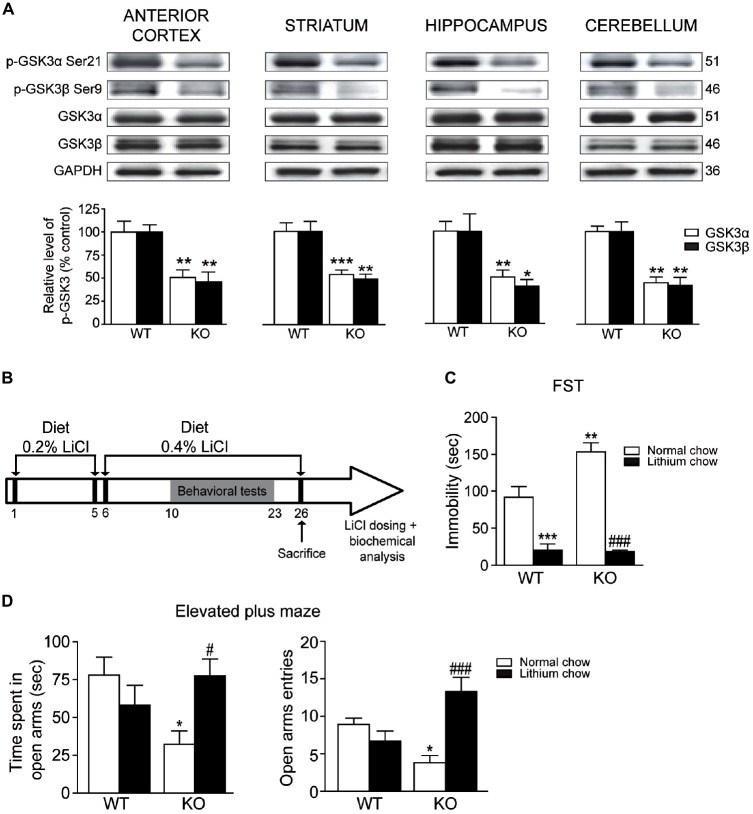
**Chronic lithium treatment reversed depressive and anxiety-like behaviors in Akt3 KO mice. (A)** Levels of phosphorylated GSK3α/β were evaluated by western blot in the anterior cortex, striatum, hippocampus and cerebellum of Akt3 KO and WT mice. The data, expressed relative to total GSK3α/β, represent the mean of relative optical density (expressed as a percentage of control values) ± SEM, *n* = 3 mice/group; triplicate experiments for each mouse/group. *p < 0.05, **p < 0.01, ***p < 0.001 vs. WT mice. **(B)** Experimental design of chronic lithium treatment. **(C)** Depressive-like behavior was evaluated by the FST after normal or lithium diet. Data express the mean time of immobility in seconds ± SEM, *n* = 6–12 mice/group. **p < 0.01, ***p < 0.001 vs. WT fed with normal chow, ^###^*p* < 0.001 vs. Akt3 KO mice fed with normal chow. **(D)** Anxiety-like behavior was evaluated in the elevated plus maze after normal or lithium diet. Data are expressed as the mean ± SEM, *n* = 10–12 mice/group. *p < 0.05 vs. WT fed with normal chow, ^#^*p* < 0.05, ^###^*p* < 0.001 vs. Akt3 KO mice fed with normal chow.

We next investigated whether the deletion of Akt3 in mice could affect GSK3, a known direct downstream target of Akt. Our western blot analysis revealed that levels of phosphorylated GSK3α/β at serine 21/9 were significantly decreased in the anterior cortex (unpaired *t*-test, GSK3α: *p* < 0.01; GSK3β: *p* < 0.01), striatum (unpaired *t*-test, GSK3α: *p* < 0.001; GSK3β: *p* < 0.01), hippocampus (unpaired *t*-test, GSK3α: *p* < 0.01; GSK3β: *p* < 0.05) and cerebellum (unpaired *t*-test, GSK3α: *p* < 0.01; GSK3β: *p* < 0.01) of Akt3 KO mice (Figure 5A). In every Akt3 KO brain structures analyzed, no difference in levels of total GSK3α/β was observed compared to WT mice.

To verify the effect of a chronic treatment on the depressive and anxiety-like phenotypes observed in Akt3 KO mice, we used the mood stabilizer lithium (Figure 5B). The serum lithium concentrations were measured in mice at the end of treatments. Regardless of mice genotype, lithium concentration achieved a mean of 0.089 ± 0.07 mM in mice fed with lithium chow, whereas levels in mice fed with normal chow were undetectable. In the FST, two-way ANOVA followed by the Bonferroni *post hoc* test revealed that chronic lithium treatment significantly decreased immobility time in Akt3 KO and WT mice (treatment, *F*_(1,19)_ = 76.67; *p* < 0.001; genotype, *F*_(1,19)_ = 6.446; *p* < 0.05; treatment × genotype, *F*_(1,19)_ = 7.338; *p* < 0.05; Figure 5C). In the elevated plus maze, two-way ANOVA showed that Akt3 KO mice treated with lithium spent more time in open arms (treatment, *F*_(1,34)_ = 0.2912; *p* > 0.05; genotype, *F*_(1,34)_ = 1.240; *p* > 0.05; treatment × genotype, *F*_(1,34)_ = 6.129; *p* < 0.05) and entered more frequently in the open arms compared to Akt3 KO fed with normal chow (treatment, *F*_(1,34)_ = 5.099; *p* < 0.05; genotype, *F*_(1,34)_ = 0.1706; *p* > 0.05; treatment × genotype, *F*_(1,34)_ = 15.20; *p* < 0.001; Figure 5D).

**Figure 6:**
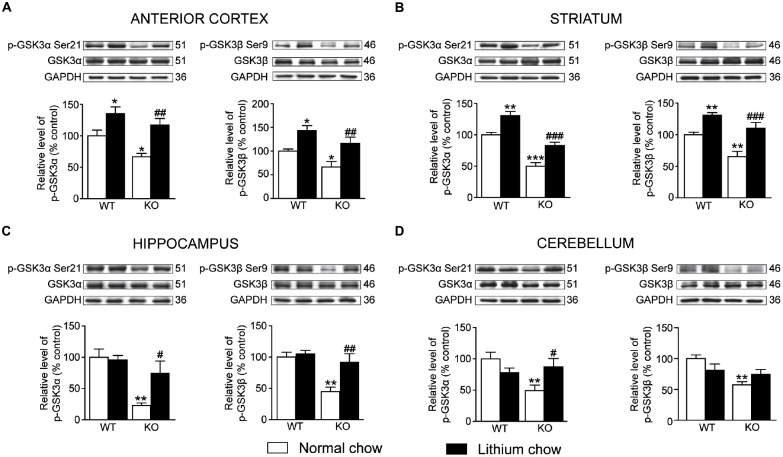
**Chronic lithium treatment increased the levels of phosphorylated GSK3α/β in Akt3 KO mice.** At the end of the chronic lithium treatment, levels of phosphorylated GSK3α/β were measured by western blot in the **(A)** anterior cortex, **(B)** striatum, **(C)** hippocampus and **(D)** cerebellum. The data, expressed relative to total GSK3α/β level, represent the mean of relative optical density of phosphorylated GSK3α/β (expressed as a percentage of control values) ± SEM, *n* = 4–8 mice/group for each condition; triplicate experiments for each mouse/group. *p < 0.05, **p < 0.01, ***p < 0.001 vs. WT fed with normal chow, ^#^*p* < 0.05, ^##^*p* < 0.01, ^###^*p* < 0.001 vs. Akt3 KO mice fed with normal chow.

Chronic lithium treatment increased the levels of phosphorylated GSK3α/β in the anterior cortex (unpaired *t*-test, GSK3α: *p* < 0.01; GSK3β: *p* < 0.01), striatum (unpaired *t*-test, GSK3α: *p* < 0.001; GSK3β: *p* < 0.001) and hippocampus (unpaired *t*-test, GSK3α: *p* < 0.05; GSK3β: *p* < 0.01) of Akt3 KO mice (Figures 6A–D). Only levels of phosphorylated GSK3α were increased in the cerebellum (unpaired *t*-test, GSK3α: *p* < 0.05; GSK3β: *p* > 0.05) of Akt3 KO mice. The lithium treatment had no effect on the levels of total GSK3α/β (Figures 6A–D).

## Discussion

The specific function of Akt3 isoform in the brain is poorly understood. Our study on Akt3 KO mice reports that Akt3 deletion is associated with normal spatial learning and memory as revealed by the Morris water maze and novel object recognition tests. In addition, our results in the rotarod, pole, wire and stepping tests provide evidence that the Akt3 KO mice do not suffer from a generalized deficit in locomotion, motor abilities and activity levels. In fact, Akt3 deletion rather evokes changes in mouse behavior reflecting psychiatric manifestations reminiscent of schizophrenia, anxiety and depression. For instance, Akt3 KO mice exhibited reduced prepulse inhibition and social novelty, two phenotypes often seen in animal models of schizophrenia (Nestler and Hyman, 2010). In particular, the prepulse inhibition is disrupted in patients suffering from schizophrenia (Braff et al., 2001) and asociality is considered to be one of the most problematic symptoms of schizophrenia (Murphy et al., 2006). Our results in two antidepressant-sensitive tests, the force swim and TSTs, and three anxiogenic tests, the open field, the elevated plus maze and the dark-light tests, suggest that deletion of Akt3 increases susceptibility to develop symptoms related to depression and anxiety. Altogether, these comprehensive behavioral manifestations propose that Akt3 signaling contributes to the normal functioning of the neural circuitry intervening in the symptoms associated with schizophrenia, depression and anxiety.

Systematic behavioral studies have never been performed on the Akt3 KO mice before. However, it is established that these mice have a reduction in brain weight resulting from a decline in both cell size and number, but preserve normal glucose homeostasis and body weights (Easton et al., 2005; Tschopp et al., 2005). Based on these observations, it is believed that Akt3 could be an important regulator of postnatal brain development (Tschopp et al., 2005). Interestingly, mood disorders such as schizophrenia and major depression showed common and distinct patterns of volumetric alteration relative to healthy individuals in a number of brain areas (Wright et al., 2000; van Tol et al., 2010; Balevich et al., 2015; Wise et al., 2016). The possibility that the observed psychiatric-like behaviors may originate from the reduced brain volume induced by Akt3 deletion is interesting, but highly speculative. A small number of clinical studies suggest that a copy number variation at the Akt3 locus could be associated with abnormal brain size. For instance, individuals with Akt3 deletions show microcephaly (Boland et al., 2007), while duplications or gain-of-function Akt3 mutations are associated with megalencephaly (Rivière et al., 2012; Wang et al., 2013; Nellist et al., 2015) and focal cortical dysplasia (Chung et al., 2014). Whether Akt3 is the only responsible gene for these witnessed clinical phenotypes is unclear. Further investigations are definitively required in order to untangle the putative brain consequence of impaired Akt3 activity. It is becoming apparent, however, that Akt3 plays a critical role in brain development and psychiatric manifestations in mice.

While the link between deletion of Akt3 isoform and psychiatric behaviors has never been established before, impaired Akt activity was previously suspected in psychiatric conditions. For example, post mortem tissue samples from patients with schizophrenia showed significant reductions of phosphorylated Akt levels in hilar neurons of the dentate gyrus and the neurogenic zone of the hippocampus (Balu et al., 2012). Akt activity is decreased in some brain regions (occipital cortex and ventral prefrontal cortex) of depressed patients (Hsiung et al., 2003; Karege et al., 2007). Phosphorylated Akt levels in the ventral tegmental area are markedly decreased in mice subjected to a depression model induced by stress, an effect reversed by fluoxetine (Krishnan et al., 2008). The study of normal physiological functions of each Akt isoform in the adult brain has been made possible recently by the generation of KO mouse models. Basic behavioral profiling of Akt1 KO mice showed that there are no overall motor, anxiety and widespread cognitive behavioral deficits, although displaying abnormal working memory involving the hippocampus (Lai et al., 2006; Balu et al., 2012). On the other hand, Akt2-deficient mice exhibit normal growth, but develop a diabetes-like syndrome with elevated fasting plasma glucose levels, elevated hepatic glucose output, and peripheral insulin resistance (Cho et al., 2001; Garofalo et al., 2003). Lately, Akt2 deletion has been associated with anxiety and depressive-like behaviors (Leibrock et al., 2013). Our data in Akt3 KO mice, showing preserved normal glucose homeostasis and body weights (Easton et al., 2005; Tschopp et al., 2005), demonstrate an endophenotype reminiscent of schizophrenia, depression and anxiety. A more methodical behavioral study using all three KO mice is definitively required in order to further understand the specific role of each isoform. It is still remarkable to note that the three Akt isoforms do not exhibit identical functions. Another evidence for this contention is the fact that these KO mice studies demonstrate no compensatory upregulation of the remaining isoforms (assessed at the mRNA or protein levels). Correspondingly, our study reveals no effect of Akt3 deletion on the protein levels of Akt1 and Akt2 in the anterior cortex, striatum, hippocampus and cerebellum of Akt3 KO mice.

Our behavioral and electrophysiological observations on Akt3 KO mice suggest that Akt3 signaling may not play such an important role in hippocampal related functions. To begin with, normal spatial navigation and recognition memory are revealed in these mice using two independent tests, the Morris water maze and the object recognition. Many studies have highlighted the implication of the hippocampus as a part of the brain responsible for spatial navigation and recognition memory (Broadbent et al., 2004; Vann and Albasser, 2011). It is therefore interesting that electrophysiological measurements achieved in the hippocampal area CA1 of Akt3 KO mice are comparable to WT mice; which correlate nicely to the behavioral data. Since Akt3 KO mice exhibit normal performances at the Morris water maze, in the novel object recognition test and in four motor abilities tests, we believe that other brain functions, like motricity and motivation, are unaltered in Akt3 KO mice. This additional evidence further underscores the existence of a brain regional specificity of Akt3 function. Levels of phosphorylated GSK3, a known direct Akt molecular target, are robustly decreased in the hippocampus of Akt3 KO mice. Lower levels of GSK3 phosphorylation is well known to lead to enhanced GSK3 kinase activity and signaling (Freland and Beaulieu, 2012). A previous study performed in knockin mice with serine-to-alanine mutations of GSK3α/β, to block inhibitory serine-phosphorylation, confirms our results (Polter et al., 2010). These GSK3 knockin mice display increased susceptibility to stress-induced depressive-like behaviors, whereas normal electrophysiological recording from the hippocampal area CA1 is shown. Altogether, these results suggest that while Akt3 deletion is associated with an endophenotype reminiscent of schizophrenia, anxiety and depression, the Akt3/GSK3 signaling has no influence in hippocampal area CA1 functions.

Akt3 deletion decreases GSK3α phosphorylation at serine 21 and GSK3β at serine 9 in every brain structure analyzed in Akt3 KO mice, suggesting that the observed phenotype could be a consequence of altered GSK3α/β phosphorylation. Since Akt can modulate over a hundred of known substrates (Manning and Cantley, 2007), we cannot exclude that the observed phenotype in Akt3 KO mice may be associated with other signaling pathways. Nevertheless, impaired GSK3α/β activity has been previously recognized to play a role in psychiatric conditions (Li and Jope, 2010). For instance, previous publications have shown that anxiety and depressive-like behaviors are associated with decreased brain levels of phosphorylated GSK3α/β (Polter et al., 2010; Beurel et al., 2011). Notably, in our experiments, the chronic lithium treatment known to amplified GSK3α phosphorylation at serine 21 and GSK3β at serine 9 (Mines et al., 2010; Liu et al., 2011), is able to reverse the behavioral signs of anxiety and depression observed in Akt3 KO mice. Besides, we observe that lithium is returning back to control value the decreased phosphorylated levels of GSK3α/β in the Akt3 KO mice. Since lithium plasma concentrations measured were within the therapeutic range (0.4–1.2 mEq/L) and no relevant adverse effects were observed in mice fed with lithium (Malhi and Tanious, 2011), we believe the administered doses of lithium are clinically relevant in our experimentations. The mechanism by which lithium amplified GSK3α/β phosphorylation in Akt3 KO mice was unfortunately not assessed by our study. Lithium is a competitive inhibitor of magnesium and can directly inhibit Mg^2+^-ATP-dependent catalytic activity of GSK3. Alternatively, indirect GSK3 inhibition can be induced through the inhibition of the protein phosphatase-2A or by increasing Akt activity (Pan et al., 2011; Freland and Beaulieu, 2012). In that regards, it was demonstrated that Akt1 isoform is essential for lithium to modulate mood-related behaviors in mice (Pan et al., 2011). Protein levels of Akt1 are not affected in the Akt3 KO mice but it is still possible that the observed amplified GSK3α/β phosphorylation is caused by an elevation of Akt1 activity. Additional biochemical experiments are definitively required to confirm this hypothesis. However, our data suggests that mechanisms underlying the lithium therapeutic effects are intact in Akt3 KO mice. They also confirm the implication of Akt3/GSK3α/β pathway in the depressive and anxiety-like behaviors seen in these mice.

It is noteworthy that recent human genetic studies are in accord with our data and suggest that impaired Akt3 is associated with schizophrenia, depression and anxiety endophenotypes. In particular, Akt1 was originally identified as a potential schizophrenia susceptibility gene (Emamian et al., 2004). However, a recent multi-stage schizophrenia genome-wide association study of up to 36,989 cases and 113,075 controls did not reproduce this finding (Schizophrenia Working Group of the Psychiatric Genomics Consortium, 2014). They reported instead that only Akt3 represents a potential contributor to schizophrenia with a high risk factor of 64 out of 108 conservatively defined loci that meet genome-wide significance. Another argument is the fact that FXR1P, a direct molecular substrate of GSK3, has also been identified as a high schizophrenia risk factor (26/108) in this study. Notably, the study establishing FXR1P as a GSK3 substrate has also demonstrated the importance of these proteins interaction in the regulation of mood and emotion processing (Del’Guidice et al., 2015). Altogether, our data paired with these recent evidences raise the interesting possibility that Akt3 might play a pivotal role in human brain pathologies such as schizophrenia, depression and anxiety.

In conclusion, modeling of human neuropsychiatric disorders in rodents is extremely challenging. Progress in understanding key molecular target in order to develop new therapeutics would certainly benefit to this research field. Therefore, the involvement of the Akt3 isoform in mice behaviors related to schizophrenia and mood disorders is novel and highly relevant. We believe that further investigation on the Akt3 isoform could lead to a better understanding of the molecular basis implicated in neuropsychiatric diseases.

## Author Contributions

YB and MC designed the study. YB conducted this research. GB contributed for the behavioral experiments. M-ÉL-L conducted the electrophysiology experiments. YB and MC wrote the manuscript. EA provided the Akt3 KO mice. MC and GM contributed to the conceptual frame of the study and edited the manuscript.

## Conflict of Interest Statement

The authors declare that the research was conducted in the absence of any commercial or financial relationships that could be construed as a potential conflict of interest.
